# Reply: Expression of oestrogen receptor beta proteins in human breast cancer biopsies

**DOI:** 10.1038/sj.bjc.6600535

**Published:** 2002-09-04

**Authors:** P T K Saunders, W R Miller

**Affiliations:** MRC Human Reproductive Sciences Unit, The University of Edinburgh Academic Centre, Edinburgh EH16 4SB, UK; Breast Cancer Research Unit, Western General Hospital, Edinburgh EH4 2XU, UK

## Abstract

*British Journal of Cancer* (2002) **87**, 688–689. doi:10.1038/sj.bjc.6600535 www.bjcancer.com

© 2002 Cancer Research UK

## Sir

We welcome the comments of Speirs and her co-workers concerning the variation and expression of ERβ protein in breast cancer tissues as reported by ourselves (Saunders *et al*, 2002a) and Skliris *et al* (2001). Clearly, the differences may be caused by variations in sensitivity or specificity of the assays, or both, and it will be important to pinpoint the reason. If sensitivity (and the methodology which might lead to this is discussed below), then discrepancies may be resolved by determining appropriate cut-off values. On the other hand, differences in specificity may produce variations that are not simply related to ERβ expression. It may therefore be useful to add the following perspective.

In our laboratory we have used a polyclonal antibody raised to hinge domain of ERβ for several studies on human tissues (Critchley *et al*, 2001; Saunders *et al*, 2000). When this antibody was tested on breast tissue sections we detected expression of nuclear receptor in most sections within both normal and cancerous cells (unpublished observations). In 1998 two papers were published showing that several isoforms of ERβ can be encoded by mRNAs formed by alternative splicing at the 3′ end of the gene (Moore *et al*, 1998; Ogawa *et al*, 1998). Furthermore, Ogawa *et al* (1998) showed that the protein of one of these forms (Erβcx/ERβ2), that lacks the ability to bind oestradiol, could be detected in tissue culture cells following transfection and could blunt the response to oestrogen if co-expressed with ERα. Because the peptide used to raise our polyclonal antibody could recognise both the wildtype ERβ and this variant isoform we repeated our studies on breast tissue using a monoclonal antibody raised against the C terminus of full-length ERβ1 (Saunders *et al*, 2002a) which we have shown does not cross react with ERβcx/ERβ2 (Saunders *et al*, 2002b). Although the biological significance of the expression of ERβ variant proteins is not known it is notable that expression of ERβcx has been reported to occur in prostatic cancers (Fujimura *et al*, 2001) and we have detected expression in breast cancer biopsies (Saunders, Miller manuscript in preparation). Immunohistochemical evaluations based on antibodies the specificity of which is not well defined may therefore lead to mis-interpretation of the likelihood that a tissue will respond to oestrogens via full-length ERβ.

In our experience there are two other factors that can have an adverse affect on the quality and reliability of detection of ERβ protein namely tissue fixation and the specificity of secondary antibodies. Based on our experiences we believe poor tissue preservation (e.g. underfixation seen in the centre of large tissue fragments) and even over fixation (cross-linking of epitopes) can account for some of the variation in the detection of ERβ reported over the last few years. Western analyses can also be problematic. We have found that ERβ breaks down readily in solution and cannot withstand more than one freeze–thaw cycle, furthermore several secondary antibodies we have tried have given false positive bands, some with molecular weights close to ERβ, when tested on membranes without the addition of primary antibody.

Taking all these factors into account it is perhaps unsurprising that we are some way from deciding how the detection of ERβ should influence decisions regarding the oestrogenic responsiveness of tissues including the breast (and it is worth noting that the application of ERα required years of refinement). However, we are currently assessing the predictive value of our assay for ERβ in tumours from patients with breast cancer receiving neo-adjuvant treatment with tamoxifen.
